# Trends in predicted 10-year risk for cardiovascular diseases among patients with type 2 diabetes in Thailand, from 2014 to 2018

**DOI:** 10.1186/s12872-023-03217-8

**Published:** 2023-04-05

**Authors:** Sethapong Lertsakulbunlue, Mathirut Mungthin, Ram Rangsin, Anupong Kantiwong, Boonsub Sakboonyarat

**Affiliations:** 1 Department of Pharmacology, Phramongkutklao College of Medicine, Bangkok, 10400 Thailand; 2 Department of Parasitology, Phramongkutklao College of Medicine, Bangkok, 10400 Thailand; 3 Department of Military and Community Medicine, Phramongkutklao College of Medicine, Bangkok, 10400 Thailand

**Keywords:** Cardiovascular diseases, Cardiovascular risks, Type 2 diabetes, Thailand

## Abstract

**Background:**

Cardiovascular diseases (CVD) are the leading causes of death globally, including Thailand. Approximately one-tenth of Thai adults have type 2 diabetes (T2D), a significantly increasing CVD. Our study aimed to determine the trends of predicted 10-year CVD risk among patients with T2D.

**Methods:**

A series of hospital-based cross-sectional studies were conducted in 2014, 2015 and 2018. We included Thai patients with T2D aged 30–74-year-old without a history of CVD. The predicted 10-year risk for CVD was calculated based on Framingham Heart Study equations both with simple office-based nonlaboratory and laboratory-based. Age- and sex-adjusted means and proportions of predicted 10-year risk for CVD were calculated.

**Results:**

A total of 84,602 patients with T2D were included in the present study. The average SBP among study participants was 129.3 ± 15.7 mmHg in 2014 and rose to 132.6 ± 14.9 mmHg in 2018. Likewise, the average body mass index was 25.7 ± 4.5 kg/m^2^ in 2014 and elevated to 26.0 ± 4.8 kg/m^2^ in 2018. The age- and sex-adjusted mean of the predicted 10-year CVD risk (simple office-based) was 26.2% (95% CI: 26.1–26.3%) in 2014 and rose to 27.3% (95% CI: 27.2–27.4%) in 2018 (*p*-for trend < 0.001). While the age- and sex-adjusted mean of the predicted 10-year CVD risk (laboratory-based) ranged from 22.4–22.9% from 2014 to 2018 (*p*-for trend < 0.001). The age- and sex-adjusted prevalence of the high predicted 10-year CVD risk (simple office-based) was 67.2% (95% CI: 66.5–68.0%) in 2014 and significantly rose to 73.1% (95% CI: 72.4–73.7%) in 2018 (*p*-for trend < 0.001). Nevertheless, the age- and sex-adjusted prevalence of the high predicted 10-year CVD risk (laboratory-based) ranged from 46.0–47.4% from 2014 to 2018 (*p*-for trend = 0.405). However, among patients with available laboratory results, a significantly positive correlation was noted between predicted 10-year CVD risk, simple office-based and laboratory-based (r = 0.8765, *p-*value < 0.001).

**Conclusion:**

Our study demonstrated significant rising trends in the predicated 10-year CVD risk among Thai patients with T2D. In addition, the results empowered further improved modifiable CVD risks, especially regarding high BMI and high blood pressure.

**Supplementary Information:**

The online version contains supplementary material available at 10.1186/s12872-023-03217-8.

## Background

Cardiovascular diseases (CVD) are the leading causes of death globally. In 2019, the World Health Organization (WHO) estimated that 17.9 million lives were lost to this group of diseases each year [1]. Since 1990, the number of CVD deaths in Asia has continued to rise, the proportion of CVD deaths in total deaths increased from 23 to 35% in 1990 and 2019, respectively, and crude CVD mortality rates increased continuously among both men and women [2, 3].

Since 1991, the National Health Exam Survey (NHES) has been conducted in Thailand, and CVD risk factors such as type 2 diabetes (T2D) hyperlipidemia (DLP), hypertension (HT) and obesity prevalence were seen to increase [4, 5]. T2D is one of the most common noncommunicable diseases, developed in more than 10% of Thai adults [4, 6, 7], and approximately three-fourths of those have HT comorbidity [8]. Furthermore, a recent study in Thailand also demonstrated that Thai patients with T2D having body mass index (BMI) over 30 kg/m^2^ rose continuously from 2011 to 2018 [8].

Currently, to reflect the effects of several interventions, CVD risk assessment tools are increasingly used in large randomized controlled trials [9]. Various CV risk assessment algorithms have been developed, but their applicability to subjects with diabetes is uncertain [10, 11]. The Framingham risk score (FRS) is one of the most effective cardiovascular risk calculators used globally in clinical practice to identify and treat high-risk populations and effectively communicate risk [12, 13]. In Thailand, although a Thai CV risk score developed based on a specific career population is available, further external validation is still needed [14, 15]. To our knowledge, only one small study predicting the risk for CVD in the general population was reported in Thailand [16]. In addition, limited information demonstrated the predicted 10-year risk of CVD among Thai patients with T2D.

Herein, we calculated the predicted 10-year CVD risk among Thai patients with T2D based on the FRS equations using both simple office-based nonlaboratory and laboratory-based data with the database from the Thailand DM/HT [8]. Furthermore, we also determined trends in the predicted 10-year CVD risk in this population from 2014 to 2018. Understanding these trends may help in developing health policy and strategies to reduce CVD risk in this population.

## Methods

### Study design and subjects

In the present study, we used the data retrieved from the database: Assessment in Quality of Care among Patients Receiving a Diagnosis with Type 2 Diabetes and Hypertension Visiting Ministry of Public Health (MoPH) and Bangkok Metropolitan Administration Hospital in Thailand (Thailand DM/HT) [8] after obtaining permission of the National Health Security Office (NHSO) and the Medical Research Network of the Consortium of Thai Medial Schools (MedResNet). We then conducted a serial cross-sectional study in 2014, 2015 and 2018.

The Thailand DM/HT study is a hospital-based cross-sectional study among Thai adult patients with T2D or HT from all MoPH Hospitals, Bangkok Metropolitan Hospitals, and public and private clinics under the NHSO Program nationwide. The study design and data collection protocols of Thailand DM/HT are published [8]. A total of 33,288, 32,616 and 36,793 patients with T2D were recruited in 2014, 2015 and 2018, respectively [8].

**Fig. 1:**
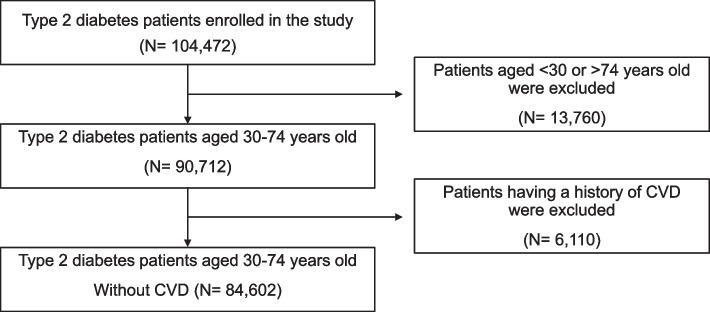
Flow of enrolled patients with T2D aged 30–74-year-old without a history of CVD

Regarding calculating the predicted 10-year CVD risk based on the FRS algorithm, we included patients with T2D aged 30 to 74 in the present study. In addition, patients with T2D having a history of CVD (coronary death, myocardial infarction, coronary insufficiency, angina, ischemic stroke, hemorrhagic stroke, transient ischemic attack, peripheral artery disease and heart failure) were excluded. Thus, our final analytic sample included 84,602 participants (Fig. 1).

### Data collection

Data were collected from the patient's medical records including baseline information, laboratory testing results and the status of diabetes complications. A standardized case report form was used for medical records, completed by a well-trained registered nurse using a standard protocol and sent to the MedResNet central data management unit in Nonthaburi, Thailand [8].

Simple office-based nonlaboratory, and laboratory-based data predicted 10-year risk for CVD, calculated using the risk algorithm derived from Framingham Heart Study (FHS) data among individuals without a history of CVD (coronary heart disease, stroke, peripheral artery disease or heart failure) [17, 18]. Simple office-based nonlaboratory data predicted 10-year risk for CVD, including variables in the algorithm, i.e., age, sex, BMI, systolic blood pressure (SBP), a history of T2D, and treatment for HT [18]. However, the laboratory-based data predicted 10-year risk for CVD, BMI substituted was total cholesterol (TC) and high-density lipoprotein (HDL) cholesterol [18]. The high predicted 10-year risk of CVD was defined as a predicted 10-year risk of CVD > 20% [18].

### Statistical analysis

All analyses were performed using StataCorp, 2021, *Stata Statistical Software: Release 17*. College Station, TX: StataCorp LLC. Frequency distribution of demographic characteristics was performed to describe the study subjects. Categorical data including sex, regions, occupation, health schemes, smoking status, history of HT and dyslipidemia were presented as percentages. Continuous variables, including age, BMI and blood pressure were presented as mean and standard deviation (SD), while laboratory blood test results were presented as median and interquartile rage (IQR). We calculated age- and sex-adjusted means or proportions of predicted 10-year risk for CVD using data for each year in 2014, 2015 and 2018. Linear regression was employed for age and sex-adjusted means and logistic regression for proportions to test the statistical significance of linear and nonlinear trends. The nonlinear trend was first tested by adding a quadratic term in the regression model. When the result was insignificant, a linear trend was tested. All statistical tests were two sided, and a *p*-value less than 0.05 was considered statistically significant.

### Ethics considerations

The study was reviewed and approved by the Institutional Review Board, RTA Medical Department (Approval no. S049h/64_Exp), in compliance with international guidelines including the Declaration of Helsinki, the Belmont Report, CIOMS Guidelines, and the International Conference on Harmonization of Technical Requirements for Registration of Pharmaceuticals for Human Use—Good Clinical Practice (ICH-GCP). Due to using secondary data, a waiver of documentation of informed consent was used, and the waiver for informed consent was granted by the Institutional Review Board, RTA Medical Department.

## Results

### Baseline characteristics of participants

A total of 84,602 patients with T2D aged between 30 and 74 without history of CVD was included in the present study. Of those participants, 26,874, 26,554 and 31,174 came from the database in 2014, 2015 and 2018, respectively. Table 1 presents the baseline characteristics of study participants. Approximately one third of the participants were males. The average age of participants ranged from 58.4 ± 9.2 to 59.3 ± 8.9 years between 2014 and 2018. In all, 32.0, 31.6, 16.2, 14.8 and 5.5% of participants resided in the northeast, central, north, south and Bangkok, respectively. Almost 80% of all participants received T2D care at community hospitals and were under the universal health coverage scheme. The mean duration of T2D diagnosis was 7.5 ± 4.8 years. The prevalence of HT comorbidity among study participants was 73.4, 75.7 and 75.3 in 2014, 2015 and 2018, respectively. The average BMI of study participants rose from 25.7 ± 4.5 kg/m^2^ in 2014 to 26.0 ± 4.7 kg/m^2^ in 2018. Similarly, the average SBP of study participants surged from 129.3 ± 15.7 mmHg in 2014 to 132.6 ± 14.9 mmHg in 2018. However, the proportion of current smokers among study participants gradually decreased from 5.0, 4.6 and 3.9% in 2014, 2015 and 2018, respectively. Only 65,173 of 84,602 participants received complete TC and HDL cholesterol information. From these individuals, we observed an increase in average HDL cholesterol level from 45 (IQR 38–54) mg/dL in 2014 to 47 (IQR 39–56) mg/dL in 2018, while TC level decreased from 184 mg/dL (IQR 158–215) in 2014 to 180 (IQR 154–210) mg/dL in 2018. Statin prescriptions increased among patients with T2D from 58.8% in 2014 to 68.5% in 2018.

**Table 1:** Demographic characteristics of participants (*n* = 84,602)

**Year**	**2014**	**2015**	**2018**
**Characteristics**	***n* = 26,874**	***n* = 26,554**	***n* = 31,174**
	**n (%)**	**n (%)**	**n (%)**
**Sex**			
Male	8079 (30.1)	8451 (31.8)	10,205 (32.7)
Female	18,795 (69.9)	18,103 (68.2)	20,969 (67.3)
**Age (years)**			
30–39	778 (2.9)	701 (2.6)	706 (2.3)
40–49	3991 (14.9)	3561 (13.4)	3737 (12.0)
50–59	9018 (33.6)	8948 (33.7)	10,154 (32.6)
60–69	9929 (36.9)	10,049 (37.8)	12,462 (40.0)
70–74	3158 (11.7)	3295 (12.4)	4115 (13.2)
Mean ± S.D	58.4 ± 9.2	58.8 ± 9.1	59.3 ± 8.9
**Geographic regions**			
North	4010 (14.9)	4262 (16.1)	5416 (17.4)
Central	7787 (29.0)	8755 (33.0)	10,172 (32.6)
Northeast	10,057 (37.4)	8100 (30.5)	8877 (28.5)
South	3593 (13.4)	3726 (14.0)	5200 (16.7)
Bangkok	1427 (5.3)	1711 (6.4)	1509 (4.8)
**Hospital level**			
Regional hospital (S/A)	1810 (6.7)	2260 (8.5)	2032 (6.5)
General hospital	3755 (14.0)	4243 (16.0)	4685 (15.0)
Community hospital	21,309 (79.3)	20,051 (75.5)	24,457 (78.4)
**Occupation**			
Agriculturist	12,040 (44.8)	10,951 (41.2)	12,389 (39.7)
Retirement	6454 (24.0)	6807 (25.6)	9199 (29.5)
Employee	4744 (17.7)	4980 (18.8)	5740 (18.4)
Private business	1417 (5.3)	1811 (6.8)	1998 (6.4)
Government officer	1116 (4.2)	1310 (4.9)	1505 (4.8)
Others	1108 (4.1)	695 (2.6)	343 (1.1)
**Health scheme**			
Univeral healthcare coverage	21,394 (79.8)	20,601 (77.6)	24,674 (79.1)
Civil servant medical benefit	3822 (14.2)	4175 (15.7)	4649 (14.9)
Social security	1248 (4.7)	1237 (4.7)	1433 (4.6)
Others	361 (1.3)	541 (2.0)	418 (1.3)
**Current smoker**	1273 (5.0)	1162 (4.6)	1201 (3.9)
**Hypertension**	19,720 (73.4)	20,096 (75.7)	23,475 (75.3)
**Dyslipidemia**	18,401 (68.5)	19,307 (72.7)	21,793 (69.9)
**Receiving anti-hypertensive medications**	19,215 (71.5)	19,351 (72.9)	22,633 (72.6)
**Receiving statin therapy**	11,411 (58.8)	12,012 (60.9)	17,825 (68.5)
**Diabetes duration (years)**			
Mean ± S.D	7.1 ± 4.6	7.5 ± 4.7	8.0 ± 5.0
**Body mass index (kg/m**^**2**^**)**			
Mean ± S.D	25.7 ± 4.5	25.9 ± 4.6	26.0 ± 4.7
**Systolic blood pressure (mmHg)**			
Mean ± S.D	129.3 ± 15.7	131.2 ± 15.8	132.6 ± 14.9
**Diastolic blood pressure (mmHg)**			
Mean ± S.D	74.6 ± 9.9	75.1 ± 10.1	75.2 ± 10.0
**Fasting plasma glucose (mg/dL)**			
n (%)^a^	24,492 (91.1)	24,721 (93.1)	29,595 (94.9)
Median (IQR)	143 (120–177)	143 (120–177)	143 (121–176)
**Hemoglobin A1c (%)**			
n (%)^a^	20,857 (77.6)	21,441 (80.7)	29,105 (93.4)
Median (IQR)	7.6 (6.6–9.1)	7.6 (6.6–9.1)	7.6 (6.6–9.0)
**LDL cholesterol (mg/dL)**			
n (%)^a^	23,498 (87.4)	23,389 (88.1)	29,282 (93.9)
Median (IQR)	106 (84–132)	105 (83–131)	102 (80–128)
**HDL cholesterol (mg/dL)**			
n (%)^a^	20,681 (77.0)	20,616 (77.6)	26,624 (85.4)
Median (IQR)	45 (38–54)	46 (38–55)	47 (39–56)
**Total cholesterol (mg/dL)**			
n (%)^a^	21,882 (81.4)	22,004 (82.9)	28,004 (89.8)
Median (IQR)	184 (158–215)	182 (156–213)	180 (154–210)

^a^have a laboratory testing, *SD* standard deviation

### Trends in mean predicted 10-year risk for CVD among patients with T2D in Thailand from 2014 to 2018

A total of 79,697 and 65,173 patients with T2D were included in the analysis to calculate the predicted 10-year CVD risk based on simple office-based nonlaboratory, and laboratory-based data, respectively. The age- and sex-adjusted and age-adjusted means of predicted 10-year risk for CVD among patients with T2D in Thailand are shown in Table 2 while Table 3 illustrates the prevalence of high predicted 10-year risk for CVD. A different proportion was noted of patients residing in each region across the survey year; we performed sensitivity analysis by including the region variable to estimate the age- sex- and region-adjusted predicted 10-year CVD risk (Supplementary File 1).

**Table 2:** Age- and sex-adjusted and age-adjusted means (%) of predicted 10-year risk for CVD

**Year**	**2014**			**2015**			**2018**			
	**n**	**mean**	**95% CI**	**n**	**mean**	**95% CI**	**n**	**mean**	**95% CI**	***p* for trends**
**Age- and sex-adjusted and age-adjusted mean (%) of projected 10-year risk for CVD (Office-based)**										
**Total**^**a**^	24,590	26.2	26.1–26.3	24,869	26.7	26.8–27.1	30,238	27.3	27.2–27.4	< 0.001^d^
**Sex**^**b**^										
Male	7286	36.0	35.7–36.2	7788	36.4	36.2–36.6	9847	36.6	36.4–36.8	0.001^d^
Female	17,304	21.7	21.6–21.9	17,081	22.7	22.5–22.8	20,391	23.2	23.0–23.3	< 0.001^d^
**Age (years)**^**c**^										
30–39	710	6.7	6.4–6.9	668	6.9	6.7–7.2	665	6.7	6.4–7.0	0.793
40–49	3636	12.8	12.6–13.0	3316	13.1	12.8–13.3	3644	13.1	12.9–13.3	0.058
50–59	8288	21.5	21.3–21.7	8428	22.0	21.8–22.2	9873	22.3	22.1–22.5	< 0.001^d^
60–69	9079	31.4	31.2–31.6	9389	32.5	32.3–32.7	12,090	33.3	33.1–33.5	< 0.001^d^
70–74	2877	40.7	40.3–41.1	3068	42.0	41.6–42.4	3966	42.3	42.0–42.7	< 0.001^d^
**Geographic region**^**a**^										
North	3844	26.2	25.9–26.5	4118	26.7	26.5–27.0	5319	26.9	26.7–27.2	0.001^d^
Central	7074	27.9	27.7–28.1	8155	28.8	28.6–29.0	9870	28.6	28.4–28.7	0.020^d^
Northeast	9480	23.9	23.8–24.1	7738	24.5	24.3–24.7	8644	25.7	25.5–25.9	< 0.001^d^
South	3355	28.2	27.9–28.5	3631	27.8	27.4–28.1	5102	27.8	27.5–28.0	0.102
Bangkok	837	28.9	28.2–29.6	1227	29.3	28.7–29.9	1303	29.7	29.1–30.2	0.137
**Age- and sex-adjusted and age-adjusted mean (%) of projected 10-year risk for CVD (Laboratory-based)**										
**Total**^**a**^	19,418	22.7	22.6–22.9	19,727	22.9	22.8–23.1	26,028	22.4	22.3–22.5	< 0.001^d^
**Sex**^**b**^										
Male	5714	33.0	32.7–33.4	6146	32.8	32.5–33.1	8436	31.6	31.4–31.9	< 0.001^d^
Female	13,704	18.1	17.9–18.2	13,581	18.5	18.3–18.6	17,592	18.3	18.1–18.4	0.379
**Age (years)**^**c**^										
30–39	579	6.2	5.9–6.5	530	6.2	5.9–6.5	576	6.0	5.7–6.3	0.158
40–49	2885	11.4	11.2–11.6	2626	11.5	11.2–11.7	3142	11.1	10.9–11.3	0.014^d^
50–59	6546	18.8	18.6–19.0	6668	19.0	18.8–19.2	8543	18.6	18.4–18.8	0.043^d^
60–69	7120	27.2	26.9–27.5	7427	27.4	27.1–27.6	10,358	27.1	26.9–27.3	0.310
70–74	2288	34.7	34.2–35.2	2476	35.2	34.7–35.7	3409	33.8	33.4–34.2	< 0.001^d^
**Geographic region**^**a**^										
North	2952	22.8	22.5–23.2	3126	22.7	22.3–23.0	4382	21.8	21.5–22.1	< 0.001^d^
Central	5759	23.2	23.0–23.5	6605	23.7	23.5–24.0	8809	22.5	22.3–22.7	< 0.001^d^
Northeast	7286	21.3	21.1–21.6	6168	21.8	21.6–22.1	7030	21.8	21.6–22.1	0.028^d^
South	2970	24.6	24.2–25.0	3102	23.4	23.0–23.8	4618	23.2	22.9–23.5	< 0.001^d^
Bangkok	451	24.6	23.6–25.6	726	23.9	23.1–24.7	1189	24.7	24.1–25.3	0.307

^a^Age- and sex adjusted mean using regression analyses, ^b^Age-adjusted mean using regression analyses, ^c^Sex-adjusted mean using regression analyses, ^d^Nonlinear trend

**Table 3:** Age- and sex-adjusted and age-adjusted prevalence of high predicted 10-year risk for CVD

**Year**	**2014**			**2015**			**2018**			*p for trend*
	**N**	**%**	**95% CI**	**N**	**%**	**95% CI**	**N**	**%**	**95% CI**	
**Age and sex-adjusted and age-adjusted percentage of high predicted 10-year risk for CVD (Office-based)**										
**Total**^**a**^	24,590	67.2	66.5–68.0	24,869	71.3	70.6–72.1	30,238	73.1	72.4–73.7	< 0.001^**d**^
**Sex**^**b**^										
Male	7286	94.7	84.2–95.2	7788	95.1	94.6–95.5	9847	94.7	94.2–95.2	0.716
Female	17,304	47.9	47.0–48.9	17,081	53.3	52.3–54.3	20,391	56.4	55.5–57.3	< 0.001^**d**^
**Age (years)**^**c**^										
30–39	710	1.2	0.6–2.3	668	1.5	0.8–2.8	665	0.8	0.4–1.8	0.135
40–49	3636	11.7	10.7–12.8	3316	12.5	11.5–13.7	3644	12.8	11.7–13.9	0.881
50–59	8288	46.1	45.0–47.2	8428	51.6	50.5–52.7	9873	52.2	51.1–53.2	< 0.001^**d**^
60–69	9079	81.5	80.6–82.3	9389	84.3	83.5–85.0	12,090	86.8	86.1–87.3	< 0.001^**d**^
70–74	2877	94.1	93.2–94.9	3068	95.0	94.2–95.8	3966	96.7	96.1–97.2	< 0.001^**d**^
**Geographic region**^**a**^										
North	3844	68.1	66.1–70.1	4118	72.9	71.0–74.6	5319	73.4	71.8–75.0	0.002^**d**^
Central	7074	75.0	73.6–76.3	8155	79.4	78.3–80.5	9870	79.1	78.0–80.1	0.002^**d**^
Northeast	9480	56.6	55.2–57.9	7738	59.8	58.3–61.2	8644	65.0	63.7–66.4	< 0.001^**d**^
South	3355	74.6	72.6–76.5	3631	73.3	71.4–75.2	5102	72.7	71.0–74.3	0.161
Bangkok	837	79.3	75.8–82.3	1227	77.2	74.2–80.0	1303	80.3	77.5–82.8	0.277
**Age and sex-adjusted and age-adjusted percentage of high predicted 10-year risk for CVD by (Laboratory-based)**										
**Total**^**a**^	19,418	46.0	45.1–46.9	19,727	47.4	46.5–48.3	26,028	46.1	45.3–46.8	0.405
**Sex**^**b**^										
Male	5714	86.5	85.5–87.4	6146	85.9	84.9–86.8	8436	83.6	82.6–84.5	< 0.001^**d**^
Female	13,704	28.7	27.8–29.5	13,581	30.5	29.6–31.4	17,592	30.1	29.3–30.9	0.094
**Age (years)**^**c**^										
30–39	579	0.5	0.1–1.9	530	0.1	0.02–0.8	576	0.2	0.03–0.8	0.114
40–49	2885	7.1	6.2–8.1	2626	7.2	6.3–8.2	3142	5.8	4.9–6.5	0.003^**d**^
50–59	6546	32.6	31.3–33.9	6668	33.8	32.5–35.1	8543	32.6	31.5–33.7	0.591
60–69	7120	70.3	69.0–71.4	7427	71.7	70.5–72.8	10,358	71.4	70.4–72.4	0.305
70–74	2288	88.3	86.7–89.7	2476	88.8	87.3–90.1	3409	88.5	87.1–89.8	0.908
**Geographic region**^**a**^										
North	2952	45.7	43.4–48.1	3126	47.2	45.0–49.5	4382	43.8	41.9–45.7	0.040^**d**^
Central	5759	50.0	48.4–51.7	6605	51.5	49.9–53.0	8809	47.9	46.5–49.2	0.002^**d**^
Northeast	7286	39.1	37.7–40.6	6168	41.7	40.1–43.3	7030	42.3	40.8–43.7	0.017^**d**^
South	2970	53.2	50.9–55.5	3102	49.2	47.0–51.5	4618	47.9	46.1–49.7	0.004^**d**^
Bangkok	451	53.1	47.5–58.7	726	53.5	49.0–58.0	1189	58.5	55.0–61.9	0.042^**d**^

^a^Age- and sex adjusted mean using regression analyses, ^b^Age-adjusted mean using regression analyses, ^c^Sex-adjusted mean using regression analyses, ^d^Nonlinear trend

#### Mean predicted 10-year risk for CVD based on simple office-based nonlaboratory data

**Fig. 2:**
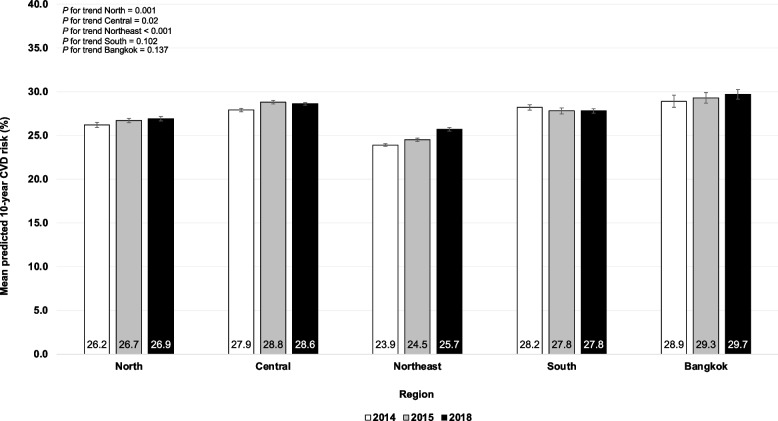
Age- and sex-adjusted mean predicted 10-year simple-office based non-laboratory CVD risk (%) among Thai patients with T2D stratified by region

The overall age and sex-adjusted mean predicted 10-year rate for CVD among patients with T2D significantly rose from 26.2% (95% CI: 26.1–26.3%) in 2014 to 27.3% (95% CI: 27.2 to 27.4%) in 2018, *p* < 0.001 for non-linear trend (Table 2). Among male participants, the age-adjusted mean predicted 10-year rate for CVD slightly rose from 36% (95% CI: 35.7 to 36.2%) in 2014 to 36.6% (95% CI: 36.4 to 36.8%) in 2018 (*p* = 0.001 for nonlinear trend). Similarly, it rose significantly from 21.7% (95% CI: 21.6 to 21.9%) in 2014 to 23.2% (95% CI: 23 to 23.3%) in 2018 among females, *p* < 0.001 for nonlinear trend. The sex-adjusted mean predicted 10-year risk for CVD tended to be higher among participants of higher age. Significantly rising trends in the sex-adjusted mean predicted 10-year risk for CVD among participants aged 50 years were observed (*p* < 0.001 for nonlinear trend). Figure 2 illustrates the age- and sex-adjusted mean of the predicted 10-year risk for CVD among patients with T2D from 2014 to 2018, stratified by regions. The age- and sex-adjusted mean of the predicted 10-year risk for CVD among study participants residing in the north, central and northeast significantly surged from 2014 to 2018 (*p* < 0.05 for nonlinear trend). Nonetheless, among study participants residing in Bangkok, it ranged consistently high from 28.9 to 29.7% between 2014 and 2018 (*p* = 0.137 for linear trend).

#### Mean predicted 10-year risk for CVD based on laboratory data

The overall age- and sex-adjusted means predicting 10-year risk for CVD among patients with T2D ranged from 22.7% in 2014 and slightly dropped to 22.4% in 2018, *p* < 0.001 for nonlinear trend (Table 2). Among male participants, the age-adjusted mean predicted 10-year risk for CVD decreased from 33% (95% CI: 32.7 to 33.4%) in 2014 to 31.6% (95% CI: 31.4 to 31.9%) in 2018 (*p* < 0.001 for nonlinear trend). However, it tended to be constant among females, ranging from 18.1 to 18.5% between 2014 and 2018 (*p* = 0.379 for linear trend). In terms of age- and sex-adjusted mean predicted 10-year risk for CVD tended to be higher among patients at higher age. In terms of region, decreasing trends were observed in the age- and sex-adjusted means predicting 10-year CVD risk among patients with T2D residing in the north, central and south from 2014 to 2018 (*p* < 0.001 for nonlinear trend). However, in Bangkok, it remained constantly high ranging from 23.9 to 24.7% between 2014 and 2018 (*p* = 0.307 for linear trend).

### Trends in prevalence of high predicted 10-year risk for CVD among patients with T2D in Thailand from 2014 to 2018

#### High predicted 10-year risk for CVD based on simple office-based nonlaboratory data

**Fig. 3:**
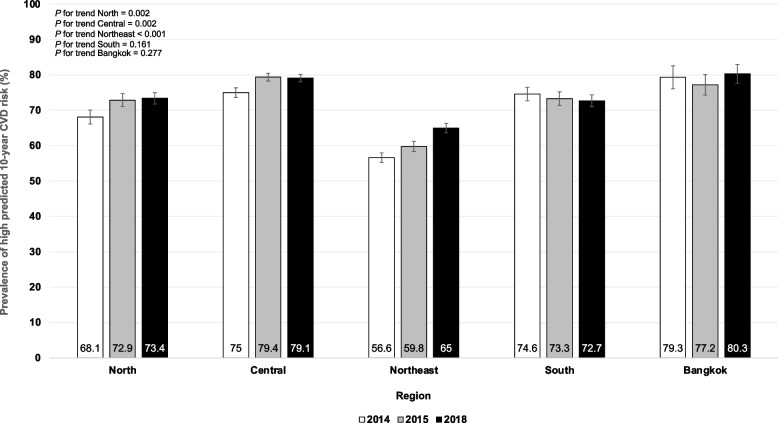
Age- and sex-adjusted prevalence of high predicted 10-year simple-office based non-laboratory CVD risk among Thai patients with T2D, stratified by region

The overall age- and sex-adjusted prevalence of high predicted 10-year risk for CVD among patients with T2D significantly surged from 67.2% (95% CI: 66.5 to 68%) in 2014 to 73.1% (95% CI: 72.4 to 73.7%) in 2018, *p* < 0.001 for nonlinear trend (Table 3). Among males, the age-adjusted prevalence of high predicted 10-year risk for CVD was constantly high, ranging from 94.7 to 95.1% between 2014 and 2018 (*p* = 0.716 for linear trend). Nevertheless, it significantly rose among females from 47.9% (95% CI: 47 to 48.9%) in 2014 to 56.4% (95% CI: 55.5 to 57.3%) in 2018 (*p* < 0.001 for nonlinear trend). Regarding age groups, the sex-adjusted prevalence of a high predicted 10-year risk for CVD tended to be higher among participants of higher age. Significantly rising trends in the sex-adjusted prevalence of high predicted 10-year risk for CVD among participants aged 50 years were observed (*p* < 0.001 for nonlinear trend). Figure 3 illustrates the age- and sex-adjusted prevalence of high predicted 10-year risk for CVD among patients with T2D from 2014 to 2018, stratified by regions. The age- and sex-adjusted prevalence of high predicted 10-year risk for CVD among study participants residing in the north, central and northeast significantly increased from 2014 to 2018 (*p* < 0.05 for nonlinear trend). Nonetheless, it remained constantly high among study participants residing in Bangkok, ranging from 77.2 to 80.3% between 2014 and 2018 (*p* = 0.277 for linear trend).

#### High predicted 10-year risk for CVD based on laboratory data

The overall age- and sex-adjusted prevalence of high predicted 10-year risk for CVD among patients with T2D was consistent, ranging from 46.0 to 47.4% between 2014 and 2018, *p* = 0.405 for linear trend (Table 3). Among males, decreasing trends in the age-adjusted prevalence of high predicted 10-year risk for CVD was observed, i.e., 86.5% (95% CI: 85.5 to 87.4%) in 2014, and dropping to 83.6% (95% CI: 82.6 to 84.5%) in 2018, *p* < 0.001 for nonlinear trend. However, the age-adjusted prevalence of high predicted 10-year risk for CVD among females tended to be constant from 2014 to 2018, *p* = 0.094 for linear trend. In terms of region, decreasing trends were observed in the age- and sex-adjusted prevalence of high predicted 10-year risk for CVD among patients with T2D residing in the north central and south from 2014 to 2018 (*p* < 0.05 for nonlinear trend). However, in Bangkok, it significantly surged from 53.1% (95% CI: 47.5 to 58.7%) in 2014 to 58.5% (95% CI: 55 to 61.9%) in 2018, *p* = 0.042 for nonlinear trend. However, the rising trend among patients with T2D residing in the northeast was observed (*p* = 0.017 for nonlinear trend).

### Correlation between predicted 10-year risk for CVD based on simple office-based nonlaboratory and laboratory data

**Fig. 4:**
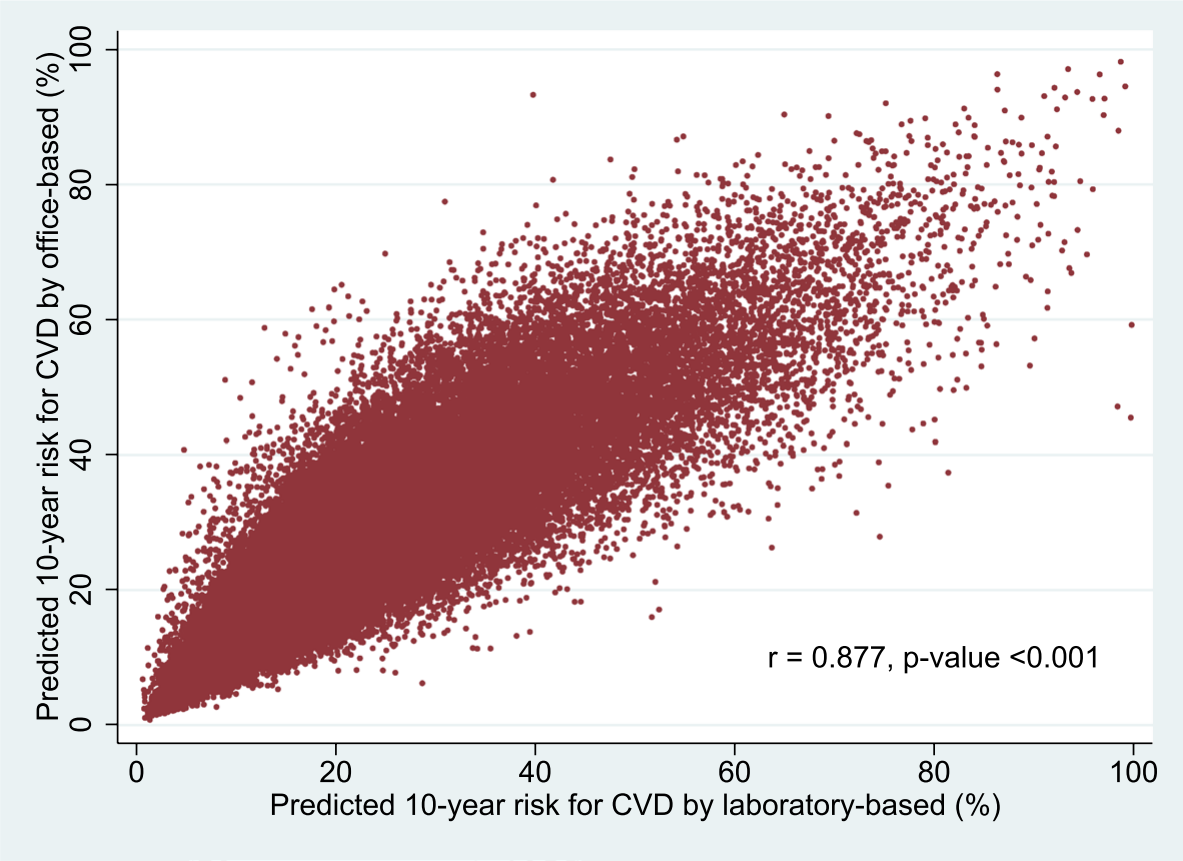
Scatter plot between predicted 10-year CVD risk, simple office-based against the laboratory-based data

In the present study, a total of 64,331 patients with T2D completed the information needed to calculate the predicted 10-year risk for CVD based on both simple office-based nonlaboratory and laboratory data. Figure 4 presents a scatter plot between predicted 10-year risk for CVD based on simple office-based nonlaboratory against the laboratory-based data. A significantly positive correlation was observed between predicted 10-year CVD risk simple office-based and laboratory-based data (*r* = 0.877, *p-*value < 0.001).

## Discussion

This nationwide serial cross-sectional study included 84,602 Thai patients with T2D aged 30 to 74 without history of CVD between 2014 and 2018. We observed changes in cardiovascular risk factors, including increasing average systolic BP and average BMI, while average TC tended to decline from 2014 to 2018. In addition, rising trends in overall age- and sex-adjusted mean and prevalence of high predicted 10-year risk for CVD by office-based data between 2014 and 2018 were observed. However, trends in the mean predicted 10-year risk for CVD using laboratory-based equations slightly decreased from 2014 to 2018, while the high predicted 10-year risk for CVD by laboratory-based data was consistent. In Thailand, a related study using a small sample size examined the predicted 10-year risk of CVD in the general Thai population based on available FHS risk scores [19]. Nevertheless, no information is available on the predicted 10-year risk of CVD among Thai patients with T2D. To our knowledge, this is the most extensive study of trends in the predicted 10-year risk of CVD among patients with T2D in Thailand.

Our study indicated that rising trends were observed in average BMI and SBP, constituting a potential risk for CVD among participants from 2014 to 2018, similar to a related report [8]. This may support our findings that significant rising trends in the age- and sex-adjusted predicted 10-year risk for CVD using an office-based approach among this study’s participants. On the other hand, when we used a laboratory-based equation to predict 10-year risk for CVD, slightly decreasing trends were observed in age- and sex-adjusted prevalence of high predicted 10-year risk for CVD from 2014 to 2018. Our findings may explain that the average TC level among patients with T2D tended to decrease from 2014 to 2018, while the average HDL cholesterol level in 2018 was more likely to be higher when compared with that in 2014. In addition, our results also revealed that statins used among study participants tended to increase from 2014 to 2018. Nevertheless, the age- and sex-adjusted prevalence of high predicted 10-year risk for CVD (> 20%) did not improve during the study period.

However, a difference in trends of predicted 10-year risk for CVD was found between both approaches during the study period. When we considered patients with T2D having completed the information to calculate the predicted 10-year risk for CVD using office-based and laboratory-based methods, a highly positive correlation between both approaches was observed. We also found that the predicted 10-year risk for CVD using an office-based equation was more likely to be high when compared with those using a laboratory-based equation which was consistent with the findings in related studies in Wales [20] and Malaysia [21]. The office-base equation required fewer laboratory measurements (BMI rather than TC and HDL cholesterol). Furthermore, obesity was confirmed to lead to the development of CVD and CVD mortality independently of other cardiovascular risk factors [22].

We found that the age-adjusted mean predicted 10-year risk for CVD among male study participants was higher than that among females over the study period, which was compatible with the related study among U.S. adults [23, 24]. In addition, our results demonstrated that more than 90% of male participants in the current study had an office-based high predicted 10-year risk for CVD, which was highly constant from 2014 to 2018. At the same time, a significantly rising trend among females was observed. These findings may be explained by the established behavioral and metabolic risk for CVD among males, which is higher than that among females in Thailand [25–28]. Notably, the present study included only patients with T2D receiving care at the hospitals; approximately one-third of the study participants were males, but the NHES in Thailand indicated that prevalence of T2D among men and women was comparable [8, 29, 30]. Gender values may partly explain the higher ratio of female patients with T2D and health-seeking behaviors in that male patients exhibited less health awareness [31, 32]. Therefore, this may have led to underestimating the predicted 10-year risk for CVD among Thai male patients with T2D.

In terms of age, well documented data indicated that higher age individuals were more likely to have a greater risk for CVD [23, 24, 33, 34]. Likewise, the present study demonstrated that sex-adjusted predicted 10-year risk for CVD was higher among higher aged patients with T2D compared with that of younger aged patients. In addition, we also found a significantly rising trend in the sex-adjusted prevalence of the high predicted 10-year risk for CVD based on an office-based approach among participants aged $\ge$ 50 from 2014 to 2018. In contrast, a related study in the U.S. presented that decreasing trends were observed in the predicted 10-year risk for CVD among people aged $\ge$ 50 from 1999 to 2010 [23]. Our results suggested that rising trends in the high predicted 10-year risk for CVD among Thai elderly patients with T2D should be recognized. Modifiable risk factors, including high blood pressure and obesity in this population, should be alleviated to prevent CVD late in life.

Sakboonyarat et al. reported that in 2013, Thai patients with T2D residing in the fourth health region located in the central area of Thailand had the highest prevalence of ischemic heart disease compared with that in other regions [25]. Our results also reported that between 2014 and 2018, the age- and sex-adjusted prevalence of high predicted 10-year risk for CVD among patients with T2D residing in Bangkok and central regions was relatively high compared with that in other regions. This finding may be explained by established behavioral risks for CVD including higher BMI and dietary patterns [4, 26], affecting BMI in the Thai populations residing in Bangkok and the central region. Although the predicted 10-year risk for CVD among patients with T2D residing in the Northeast tended to be lower than that in other regions, markedly increasing trends were also observed in the age-and sex-adjusted prevalence of high predicted 10-year risk for CVD from 2014 to 2018 among patients residing in the Northeast. These findings may be supported by the results when we performed subgroup analysis revealing that mean SBP among patients in the northeast increased substantially from 126.1 mmHg in 2014 to 131.2 mmHg in 2018. Moreover, rising trends in average BMI from 25.1 kg/m^2^ in 2014 to 25.3 kg/m^2^ in 2018 were observed among patients in the northeast.

SBP and BMI were independent predictors in the FHS risk equations, and our study indicated increasing trends in average blood pressure and BMI among patients with T2D from 2014 to 2018. Therefore, high blood pressure and high BMI, namely, modifiable behavioral and metabolic risk factors, should be alleviated to reduce the risk for CVD in this population. In addition, our study suggested that lifestyle modifications, such as a DASH diet, weight loss, increased physical activity and alcohol restriction [35–37], should be encouraged in this population. However, physical exercise should be carefully performed under physician recommendations and related guidelines [38] because excessive exercise may lead to adverse cardiovascular events [39].

The present study encountered several limitations. Firstly, the present study included only patients with T2D visiting a hospital for diabetes care and excluded patients with T2D receiving care at primary care units, accounting for approximately one half of the overall patients with T2D in Thailand. Additionally, subjects from university hospitals were excluded. Secondly, this investigation comprised a series of cross-sectional studies; longitudinal changes in the risk for CVD at an individual level could not be evaluated. Thirdly, due to the nature of an observational study, the information on some variables could have been more attainable, including BMI of 1,412 individuals (1.7%) and SBP of 138 individuals (0.26%). Therefore, regarding an extensive dataset, we did not perform imputed data; however, individuals without BMI or SBP were still included in the calculation of the risk of CVD based on laboratory equations when HDL and TC data were available. Additionally, data were missing from the patient's laboratory results (TC and HDL cholesterol), which may have affected the pattern of trends in the predicted CVD risk from 2014 to 2018. In addition, we explored characteristics between T2D with and without TC and HDL cholesterol information that may have different distributions; therefore, the interpretation of the predicted CVD risk using laboratory-based methods among this study's participants should be carefully considered.

Finally, the present study calculated the predicted 10-year risk of CVD based on FHS data which was conducted in a U.S. population. Therefore, the validity of CVD risk prediction among Thais may be relatively inaccurate. Nevertheless, the FHS CVD risk score was validated in a multiethnic Asian population retrospective cohort study, showing fairly accurate prediction among males and slightly overestimated prediction for females [40]. Additionally, the calibration study among UK-based individuals with T2D indicated that CVD risk prediction scores might not accurately identify those who experienced a CVD event in the ten years of follow-up. Therefore, interpreting the results in this study as CVD risk prediction among patients with T2D should be performed carefully [41]. Our study had considerable strengths, including extensive data for predicting the 10-year risk for CVD among Thai patients with T2D. Thus, our results provided valuable insights regarding the rising trends in this population's predicted 10-year risk for CVD, empowering increased attention to the population's healthcare.

## Conclusion

Our study demonstrated rising trends in overall age- and sex-adjusted mean and prevalence of high predicted 10-year risk for CVD by office-based method between 2014 and 2018. While trends in the mean predicted 10-year risk for CVD using laboratory-based equations slightly decreased from 2014 to 2018, the high predicted 10-year risk for CVD by laboratory-based method remained consistent. The modifiable risk factors for CVD including high SBP and high BMI in this population should be attenuated to reduce the risk for CVD in the future.

## Supplementary Information

**Additional file 1:** **Supplementary Table 1.** Age-, sex- and region- adjusted, age- and region-adjusted, and sex- and region-adjusted means (%) of projected 10-year risk for CVD. **Supplementary Table 2.** Age-, sex- and region- adjusted, age- and region-adjusted, and sex- and region-adjusted percentage of high predicted 10-year risk for CVD.

## Data Availability

Data cannot be shared publicly because the data set contains identifying information; additionally, the data belong to the Thailand DM/HT study of the MedResNet. Thus, ethics restrictions exist on the data set. Data are available from the Thai NHSO, Bangkok, Thailand (contact Sirikorn Khunsri via sirikorn@nhso.go.th) for researchers meeting the criteria to access confidential data.

## Abbreviations

NCDs: Noncommunicable diseases
CVD: Cardiovascular diseases
FHS: Framingham Heart Study
T2D: Type 2 diabetes
BMI: Body mass index
SBP: Systolic blood pressure
HT: Hypertension
CI: Confidence interval
SD: Standard deviation
IQR: Inter quartile range
