# Supplementary material for: Trends in predicted 10-year risk for cardiovascular diseases among patients with type 2 diabetes in Thailand, from 2014 to 2018
Source: BMC Cardiovasc Disord. 2023 Apr 5;23:183. doi: 10.1186/s12872-023-03217-8 (PMC10077638; doi:10.1186/s12872-023-03217-8)
Supplement: Supplementary file 1 — Additional file 1: Supplementary Table 1. Age-, sex- and region- adjusted, age- and region-adjusted, and sex- and region-adjusted means (%) of projected 10-year risk for CVD. Supplementary Table 2. Age-, sex- and region- adjusted, age- and region-adjusted, and sex- and region-adjusted percentage of high predicted 10-year risk for CVD. [file 12872_2023_3217_MOESM1_ESM.docx]

**Trends in predicted 10-year risk for cardiovascular diseases among patients with type 2 diabetes in Thailand, from 2014 to 2018**

Sethapong Lertsakulbunlue^1^, Mathirut Mungthin^2^, Ram Rangsin^3^, Anupong Kantiwong^1^ _,_ *Boonsub Sakboonyarat^3^

^1^Department of Pharmacology, Phramongkutklao College of Medicine, Bangkok 10400, Thailand.

^2^Department of Parasitology, Phramongkutklao College of Medicine, Bangkok 10400, Thailand.

^3^Department of Military and Community Medicine, Phramongkutklao College of Medicine, Bangkok 10400, Thailand.

| **Supplementary table 1. Age-, sex- and region- adjusted, age- and region-adjusted, and sex- and region-adjusted means (%) of projected 10-year risk for CVD** | | | | | | | | | | |
| --- | --- | --- | --- | --- | --- | --- | --- | --- | --- | --- |
| **Year** | **2014** | | | **2015** | | | **2018** | | |  |
|  | n | means | 95% CI | n | means | 95% CI | n | % | 95% CI | *p* for trends |
| **Age-, sex- and region-adjusted, age- and region-adjusted, and sex- and region-adjusted means (%) of projected 10-year risk for CVD (Simple Office-based non-laboratory)** | | | | | | | | | | |
| **Total^a^** | 24590 | 26.2 | 26.1-26.3 | 24869 | 27.0 | 26.8-27.1 | 30238 | 27.3 | 27.2-27.5 | <0.001^d^ |
| **Sex^b^** |  |  |  |  |  |  |  |  |  |  |
| Male | 7286 | 36.0 | 35.7-36.2 | 7788 | 36.4 | 36.2-36.6 | 9847 | 36.6 | 36.4-36.8 | 0.078 |
| Female | 17304 | 21.7 | 21.6-21.9 | 17081 | 22.7 | 22.5-22.8 | 20391 | 23.2 | 23.0-23.3 | <0.001^d^ |
| **Age (years) ^c^** |  |  |  |  |  |  |  |  |  |  |
| 30-39 | 710 | 6.7 | 6.4-6.9 | 668 | 7.0 | 6.7-7.2 | 665 | 6.7 | 6.4-7.0 | 0.375 |
| 40-49 | 3636 | 12.8 | 12.6-13.0 | 3316 | 13.1 | 12.8-13.3 | 3644 | 13.1 | 12.9-13.3 | 0.391 |
| 50-59 | 8288 | 21.5 | 21.3-21.7 | 8428 | 22.0 | 21.8-22.2 | 9873 | 22.3 | 22.1-22.5 | <0.001^d^ |
| 60-69 | 9079 | 31.4 | 31.2-31.6 | 9389 | 32.5 | 32.3-32.7 | 12090 | 33.3 | 33.1-33.5 | <0.001^d^ |
| 70-74 | 2877 | 40.7 | 40.3-41.1 | 3068 | 42.0 | 41.6-42.4 | 3966 | 42.3 | 42.0-42.7 | <0.001^d^ |
| **Geographic region^e^** | |  |  |  |  |  |  |  |  |  |
| North | 3844 | 26.2 | 25.9-26.5 | 4118 | 26.7 | 26.5-27.0 | 5319 | 26.9 | 26.7-27.2 | 0.001 ^d^ |
| Central | 7074 | 27.9 | 27.7-28.1 | 8155 | 28.8 | 28.6-29.0 | 9870 | 28.6 | 28.4-28.7 | 0.020 ^d^ |
| Northeast | 9480 | 23.9 | 23.8-24.1 | 7738 | 24.5 | 24.3-24.7 | 8644 | 25.7 | 25.5-25.9 | <0.001^d^ |
| South | 3355 | 28.2 | 27.9-28.5 | 3631 | 27.8 | 27.4-28.1 | 5102 | 27.8 | 27.5-28.0 | 0.102 |
| Bangkok | 837 | 28.9 | 28.2-29.6 | 1227 | 29.3 | 28.7-29.9 | 1303 | 29.7 | 29.1-30.2 | 0.137 |
| **Age-, sex- and region- adjusted, age- and region-adjusted, and sex- and region-adjusted means (%) of projected 10-year risk for CVD (Laboratory-based)** | | | | | | | | | | |
| **Total^a^** | 19418 | 22.7 | 22.6-22.9 | 19727 | 22.9 | 22.8-23.1 | 26028 | 22.4 | 22.3-22.5 | <0.001^d^ |
| **Sex^b^** |  |  |  |  |  |  |  |  |  |  |
| Male | 5714 | 33.0 | 32.7-33.4 | 6146 | 32.8 | 32.5-33.1 | 8436 | 31.6 | 31.4-31.9 | <0.001^d^ |
| Female | 13704 | 18.1 | 17.9-18.2 | 13581 | 18.5 | 18.3-18.6 | 17592 | 18.3 | 18.1-18.4 | 0.643 |
| **Age (years)^c^** |  |  |  |  |  |  |  |  |  |  |
| 30-39 | 579 | 6.2 | 5.9-6.5 | 530 | 6.2 | 5.9-6.5 | 576 | 6.0 | 5.7-6.3 | 0.061 |
| 40-49 | 2885 | 11.4 | 11.2-11.6 | 2626 | 11.5 | 11.2-11.7 | 3142 | 11.1 | 10.9-11.3 | 0.002^d^ |
| 50-59 | 6546 | 18.8 | 18.6-19.0 | 6668 | 19.0 | 18.8-19.2 | 8543 | 18.6 | 18.4-18.8 | 0.006^d^ |
| 60-69 | 7120 | 27.2 | 26.9-27.5 | 7427 | 27.4 | 27.1-27.6 | 10358 | 27.1 | 26.9-27.3 | 0.119 |
| 70-74 | 2288 | 34.7 | 34.2-35.2 | 2476 | 35.2 | 34.7-35.7 | 3409 | 33.8 | 33.4-34.2 | <0.001^d^ |
| **Geographic region^e^** | |  |  |  |  |  |  |  |  |  |
| North | 2952 | 22.8 | 22.5-23.2 | 3126 | 22.7 | 22.3-23 | 4382 | 21.8 | 21.5-22.1 | <0.001^d^ |
| Central | 5759 | 23.2 | 23.0-23.5 | 6605 | 23.7 | 23.5-24 | 8809 | 22.5 | 22.3-22.7 | <0.001^d^ |
| Northeast | 7286 | 21.3 | 21.1-21.6 | 6168 | 21.8 | 21.6-22.1 | 7030 | 21.8 | 21.6-22.1 | 0.028^d^ |
| South | 2970 | 24.6 | 24.2-25.0 | 3102 | 23.4 | 23.0-23.8 | 4618 | 23.2 | 22.9-23.5 | <0.001^d^ |
| Bangkok | 451 | 24.6 | 23.6-25.6 | 726 | 23.9 | 23.1-24.7 | 1189 | 24.7 | 24.1-25.3 | 0.307 |
| ^a^Age-, sex- and region-adjusted mean using regression analyses, ^b^Age- and region-adjusted mean using regression analyses, ^c^Sex- and region-adjusted mean using regression analyses, ^d^Nonlinear trend.  ^e^Age- and sex-adjusted mean using regression analyses | | | | | | | | | | |

| **Supplementary table 2. Age-, sex- and region- adjusted, age- and region-adjusted, and sex- and region-adjusted percentage of high predicted 10-year risk for CVD** | | | | | | | | | | |
| --- | --- | --- | --- | --- | --- | --- | --- | --- | --- | --- |
| **Year** | **2014** | | | **2015** | | | **2018** | | | ***p for trend*** |
|  | N | % | 95% CI | N | % | 95% CI | N | % | 95% CI |  |
| **Age-, sex- and region- adjusted, age- and region-adjusted, and sex- and region-adjusted percentage of high predicted 10-year risk for CVD (Simple Office-based non-laboratory)** | | | | | | | | | | |
| **Total^a^** | 24590 | 67.2 | 66.5-68.0 | 24869 | 71.3 | 70.6-72.1 | 30238 | 73.1 | 72.4-73.7 | <0.001**^d^** |
| **Sex^b^** |  |  |  |  |  |  |  |  |  |  |
| Male | 7286 | 94.7 | 94.2-95.2 | 7788 | 95.1 | 94.6-95.5 | 9847 | 94.7 | 94.3-95.2 | 0.196 |
| Female | 17304 | 47.9 | 47.0-48.9 | 17081 | 53.3 | 52.3-54.3 | 20391 | 56.4 | 55.5-57.3 | <0.001**^d^** |
| **Age (years) ^c^** |  |  |  |  |  |  |  |  |  |  |
| 30-39 | 710 | 0.6 | 0.2-1.4 | 668 | 0.7 | 0.3-1.7 | 665 | 0.4 | 0.1-1.0 | 0.104 |
| 40-49 | 3636 | 8.8 | 7.9-9.8 | 3316 | 9.2 | 8.3-10.3 | 3644 | 8.8 | 8.0-9.8 | 0.522 |
| 50-59 | 8288 | 48.3 | 47.1-49.5 | 8428 | 53.2 | 52.0-54.4 | 9873 | 53.9 | 52.8-55.0 | <0.001**^d^** |
| 60-69 | 9079 | 87.5 | 86.7-88.3 | 9389 | 89.4 | 88.7-90.1 | 12090 | 91.2 | 90.6-91.8 | <0.001**^d^** |
| 70-74 | 2877 | 98.3 | 96.9-99.1 | 3068 | 98.6 | 97.4-99.3 | 3966 | 99.1 | 98.2-99.5 | <0.001**^d^** |
| **Geographic region^e^** |  |  |  |  |  |  |  |  |  |  |
| North | 3844 | 68.1 | 66.1-70.1 | 4118 | 72.9 | 71.0-74.6 | 5319 | 73.4 | 71.8-75 | 0.002 **^d^** |
| Central | 7074 | 75.0 | 73.6-76.3 | 8155 | 79.4 | 78.3-80.5 | 9870 | 79.1 | 78-80.1 | 0.002 **^d^** |
| Northeast | 9480 | 56.6 | 55.2-57.9 | 7738 | 59.8 | 58.3-61.2 | 8644 | 65.0 | 63.7-66.4 | <0.001 **^d^** |
| South | 3355 | 74.6 | 72.6-76.5 | 3631 | 73.3 | 71.4-75.2 | 5102 | 72.7 | 71-74.3 | 0.161 |
| Bangkok | 837 | 79.3 | 75.8-82.3 | 1227 | 77.2 | 74.2-80 | 1303 | 80.3 | 77.5-82.8 | 0.277 |
| **Age-, sex- and region- adjusted, age- and region-adjusted, and sex- and region-adjusted percentage of high predicted 10-year risk for CVD (Laboratory-based)** | | | | | | | | | | |
| **Total^a^** | 19418 | 46.0 | 45.1-46.9 | 19727 | 47.5 | 46.6-48.3 | 26028 | 46.1 | 45.3-46.8 | 0.071 |
| **Sex^b^** |  |  |  |  |  |  |  |  |  |  |
| Male | 5714 | 86.5 | 85.5-87.4 | 6146 | 85.9 | 84.9-86.8 | 8436 | 83.6 | 82.6-84.5 | <0.001**^d^** |
| Female | 13704 | 28.7 | 27.8-29.5 | 13581 | 30.5 | 29.6-31.4 | 17592 | 30.1 | 29.3-30.9 | 0.491 |
| **Age (years) ^c^** |  |  |  |  |  |  |  |  |  |  |
| 30-39 | 579 | 0.4 | 0.1-1.7 | 530 | 0.1 | 0.0-0.7 | 576 | 0.1 | 0.0-0.7 | 0.128 |
| 40-49 | 2885 | 7.1 | 6.2-8.1 | 2626 | 7.2 | 6.3-8.2 | 3142 | 5.7 | 4.9-6.5 | 0.001**^d^** |
| 50-59 | 6546 | 32.6 | 31.3-33.9 | 6668 | 33.8 | 32.5-35.1 | 8543 | 32.5 | 31.4-33.7 | 0.265 |
| 60-69 | 7120 | 70.3 | 69.0-71.4 | 7427 | 71.7 | 70.5-72.8 | 10358 | 71.4 | 70.4-72.4 | 0.736 |
| 70-74 | 2288 | 88.3 | 86.7-89.7 | 2476 | 88.8 | 87.3-90.1 | 3409 | 88.5 | 87.1-89.8 | 0.882 |
| **Geographic region^e^** |  |  |  |  |  |  |  |  |  |  |
| North | 2952 | 45.7 | 43.4-48.1 | 3126 | 47.2 | 45-49.5 | 4382 | 43.8 | 41.9-45.7 | 0.040 **^d^** |
| Central | 5759 | 50.0 | 48.4-51.7 | 6605 | 51.5 | 49.9-53 | 8809 | 47.9 | 46.5-49.2 | 0.002 **^d^** |
| Northeast | 7286 | 39.1 | 37.7-40.6 | 6168 | 41.7 | 40.1-43.3 | 7030 | 42.3 | 40.8-43.7 | 0.017 **^d^** |
| South | 2970 | 53.2 | 50.9-55.5 | 3102 | 49.2 | 47-51.5 | 4618 | 47.9 | 46.1-49.7 | 0.004 **^d^** |
| Bangkok | 451 | 53.1 | 47.5-58.7 | 726 | 53.5 | 49-58 | 1189 | 58.5 | 55-61.9 | 0.042 **^d^** |
| ^a^Age-, sex- and region-adjusted mean using regression analyses, ^b^Age- and region-adjusted mean using regression analyses, ^c^Sex- and region-adjusted mean using regression analyses, ^d^Nonlinear trend. ^e^Age- and sex-adjusted mean using regression analyses | | | | | | | | | | |
